# 

**Published:** 1846-03-01

**Authors:** 


### MEDICAL JOURNAL.

EDITED BY

AUSTIN FLINT, M. D.

**Vol. 1.** **BUFFALO, MARCH, 1846.** **No. 10.**

CONTENTS:

ORIGINAL COMMUNICATIONS.

Ricord on Venereal Diseases. By the Editor. - - - - - - - - - - P. 218
Notes of an European Tour. No. 9. By F. H. HAMILTON, M. D., Prof. of Surgery in Geneva Medical College. - - - - - - - - - - - - - - 225

EDITORIAL, MEDICAL INTELLIGENCE, ETC.

Medical Reform. - - - - - - - - - - - - - - - - - - 229

On the effects of Conium Maculatum.—Spurious Medicines. - - - - - - 232

A new Remedy for Gout and Rheumatism. - - - - - - - - - - - - 234

Principles of Forensic Medicine, by Wm. A. Guy, M. B., Cantab, etc. Edited by C. A. Lee, M. D., &c. - - - - - - - - - - - - - - - - 236

Appointment of Delegates to the National Convention. - - - - - - - 237

Fictitious Catalogues of Medical Students.—Hostility to Medical Schools. - - 238

Judicial Homicide. - - - - - - - - - - - - - - - - 239

Eau Brocchieri. - - - - - - - - - - - - - - - - - 240

Items. - - - - - - - - - - - - - - - - - - - - 240

Prospectus of Second Volume of the Medical Journal—Cover, page 2.

To Readers and Correspondents—Cover, page 3.

BUFFALO:
JEWETT, THOMAS & CO., PRINTERS AND PUBLISHERS,
Exchange Buildings, Third Story, Main Street.

